# Implementation of Inpatient Electronic Consultations During the COVID-19 Crisis and Its Sustainability Beyond the Pandemic: Quality Improvement Study

**DOI:** 10.2196/55623

**Published:** 2024-05-16

**Authors:** Anna S Aledia, Amish A Dangodara, Aanya A Amin, Alpesh N Amin

**Affiliations:** 1 Department of Medicine & Hospital Medicine University of California, Irvine Orange, CA United States

**Keywords:** COVID-19, electronic consultation, eConsult, e-consult, inpatient, consultations, pandemic, infectious, novel coronavirus, coronavirus, patients, patient, staff, health care, health care providers, tool, tools, effectiveness

## Abstract

**Background:**

Limiting in-person contact was a key strategy for controlling the spread of the highly infectious novel coronavirus (COVID-19). To protect patients and staff from the risk of infection while providing continued access to necessary health care services, we implemented a new electronic consultation (e-consult) service that allowed referring providers to receive subspecialty consultations for patients who are hospitalized and do not require in-person evaluation by the specialist.

**Objective:**

We aimed to assess the impact of implementing e-consults in the inpatient setting to reduce avoidable face-to-face referrals during the COVID-19 pandemic.

**Methods:**

This quality improvement study evaluated all inpatient e-consults ordered from July 2020 to December 2022 at the University of California Irvine Medical Center. The impact of e-consults was assessed by evaluating use (eg, number of e-consults ordered), e-consult response times, and outcome of the e-consult requests (eg, resolved electronically or converted to the in-person evaluation of patient).

**Results:**

There were 1543 inpatient e-consults ordered across 11 participating specialties. A total of 53.5% (n=826) of requests were addressed electronically, without the need for a formal in-person evaluation of the patient. The median time between ordering an e-consult and a specialist documenting recommendations in an e-consult note was 3.7 (IQR 1.3-8.2) hours across all specialties, contrasted with 7.3 (IQR 3.6-22.0) hours when converted to an in-person consult (*P*<.001). The monthly volume of e-consult requests increased, coinciding with surges of COVID-19 cases in California. After the peaks of the COVID-19 crisis subsided, the use of inpatient e-consults persisted at a rate well above the precrisis levels.

**Conclusions:**

An inpatient e-consult service was successfully implemented, resulting in fewer unnecessary face-to-face consultations and significant reductions in the response times for consults requested on patients who are hospitalized and do not require an in-person evaluation. Thus, e-consults provided timely, efficient delivery of inpatient consultation services for appropriate problems while minimizing the risk of direct transmission of the COVID-19 virus between health care providers and patients. The service also demonstrated its value as a tool for effective inpatient care coordination beyond the peaks of the pandemic leading to the sustainability of service and value.

## Introduction

When the novel coronavirus (COVID-19), the disease caused by SARS-CoV-2, began to quickly spread around the world, the high transmissibility of this disease urged health care systems to explore alternatives to face-to-face interactions that would reduce the risk of exposure for both the patient and the provider. Electronic consultations (e-consults) are asynchronous, non–face-to-face, provider-to-provider exchanges that have been shown to improve patient access to specialty care for appropriate referral problems that do not require an in-person evaluation of the patient by the specialist [1-3]. The rapid rise in COVID-19 cases induced a demand for the adoption of e-consult services and triggered an increase in the use of e-consults [4]. Although its use in the outpatient setting is well established [5,6], e-consults in the inpatient arena are relatively new.

As the only academic health system in the sixth largest county in the United States, University of California Irvine (UCI) Health has been a leader in the advancement of telehealth technologies that expand access to care and improve health care efficiency and resource use [7]. UCI already has a well-developed e-consults program in the ambulatory setting [8], and to complement this existing service, we expanded e-consults to patients who were hospitalized to further help reduce in-person contacts between consulting providers and patients, thereby minimizing disease transmission and conserving scarce personal protective equipment (PPE) during the COVID-19 crisis. Other health systems have implemented similar e-consult services for inpatients, but provider use of e-consults was temporary in response to the pandemic, favoring in-person consultative care instead [9]; inpatient e-consults were offered by only a single specialty consulting service [10,11]; and use cases involved early inpatient e-consult models [12,13]. In this study, we describe our rapid implementation of inpatient e-consults in multiple specialties and its sustained use beyond the peaks of the pandemic.

## Methods

### Setting

The inpatient e-consult service was implemented at the UCI Medical Center, a 478-bed acute care hospital providing tertiary and quaternary care, ambulatory and specialty medical clinics, behavioral health care, and rehabilitation services. Located in Orange County, California, it serves a diverse population of close to 4 million persons with broad health care needs. With more than 500 specialty and primary care physicians, UCI offers a full scope of acute and general care services. It is also the primary teaching location for UCI medical and nursing students, medical residents, and fellows, and it is home to Orange County’s only adult level 1 and pediatric level 2 trauma centers, a National Cancer Institute–designated comprehensive cancer center, a regional burn center, the county’s only hematopoietic stem cell and bone marrow transplant program, and the region’s only high-risk perinatal and neonatal program and maternal-fetal transport system. In winter 2020, UCI Medical Center opened a temporary mobile field hospital that added up to 50 acute care beds in response to a surge of patients with COVID-19.

### Implementation

The design and implementation of inpatient e-consults were guided by a steering committee, which included the Chair of the Department of Medicine and Executive Director of Hospital Medicine (who was the lead to design and develop e-consults at the UCI), a clinical informaticist, specialty physician leads, an IT build team, representatives from the Compliance and Privacy Office and Physicians Billing Group, and a project manager. Early on, members of this committee engaged UCI leadership to affirm support for the new service and obtain the IT resources needed to build the inpatient e-consults workflow. Regular steering committee meetings were established to discuss the design of the inpatient e-consults workflow and develop a process for provider reimbursement or credit. Prior to the go-live, the inpatient e-consult service was publicized by members of the steering committee through email communications with house staff. Steering committee members also hosted Zoom training (Zoom Technologies) and orientation sessions with participating consulting services, and they distributed tip sheets summarizing the steps to complete the requesting provider and responding consultant workflows.

**Figure 1:**
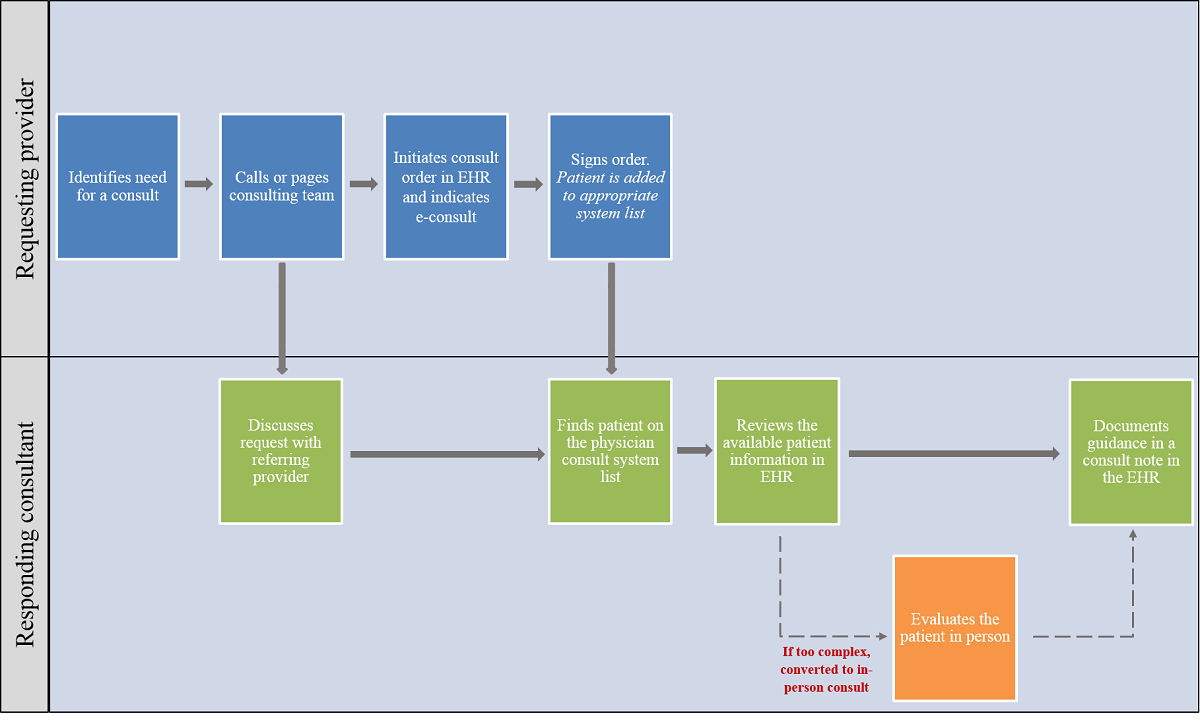
Workflows for requesting and responding to inpatient e-consults. e-consult: electronic consultation; EHR: electronic health record.

Our IT team was able to efficiently implement our inpatient e-consult service by designing workflows similar to those for traditional in-person consults. Thus, the processes for requesting and responding to inpatient e-consults were not unfamiliar to providers (Figure 1). To request an e-consult, the inpatient service or team places a consult order in the electronic health record (EHR), indicating that the request is for an e-consult. The patient is then added to the physician e-consults system list of the appropriate specialty. A follow-up call or page is also sent to the specialty by the requesting team to alert the inpatient consulting team of the e-consult and, if necessary, provide them with any additional details. To respond to the e-consult request, the inpatient consulting team reviews the relevant clinical information available in the EHR and documents their assessment and recommendations in a consult note. If the case is deemed too complex to be addressed electronically, the consulting team converts the e-consult to a traditional in-person consultation and the patient is examined before documenting guidance in the EHR. The requesting provider and responding consultant are each credited with 0.7 work relative value units (a measure of the provider’s time and effort required to perform the service) for every completed e-consult that does not result in an in-person evaluation of the patient by the consulting service, while usual billing or relative value unit credit applies for in-person consultations.

### Measurement and Analysis

We conducted a retrospective evaluation of all inpatient e-consults ordered at the UCI Medical Center from implementation in July 2020 to December 2022 to assess use, outcomes, and response times. Use was tracked by examining the volume of e-consults ordered per specialty over the 2.5-year period and comparing it with the volume of traditional in-person consults ordered for the specialties offering inpatient e-consults. To assess outcomes, we categorized the result of each e-consult order as either “resolved electronically” if the consulting team addressed the request without a face-to-face evaluation of the patient or “converted to in-person” if the consulting team deemed the case too complex and the patient required a physical examination. The response time was defined as the interval between the documented consult order in the EHR and the consulting team filing recommendations in a consult note. We calculated the median response time and the IQR in hours for each specialty and performed nonparametric Mann-Whitney *U* tests in SPSS (version 28; IBM Corp) to compare the median response times of requests resolved electronically and converted to in person. All *P* values were 2-tailed, and *P*<.05 was considered statistically significant.

### Ethical Considerations

Our implementation and retrospective analysis of the inpatient e-consults service constituted as quality improvement activities and not human subjects research. Thus, our study did not require institutional review board review. This study followed the Standards for Quality Improvement Reporting Excellence guidelines.

## Results

**Figure 2:**
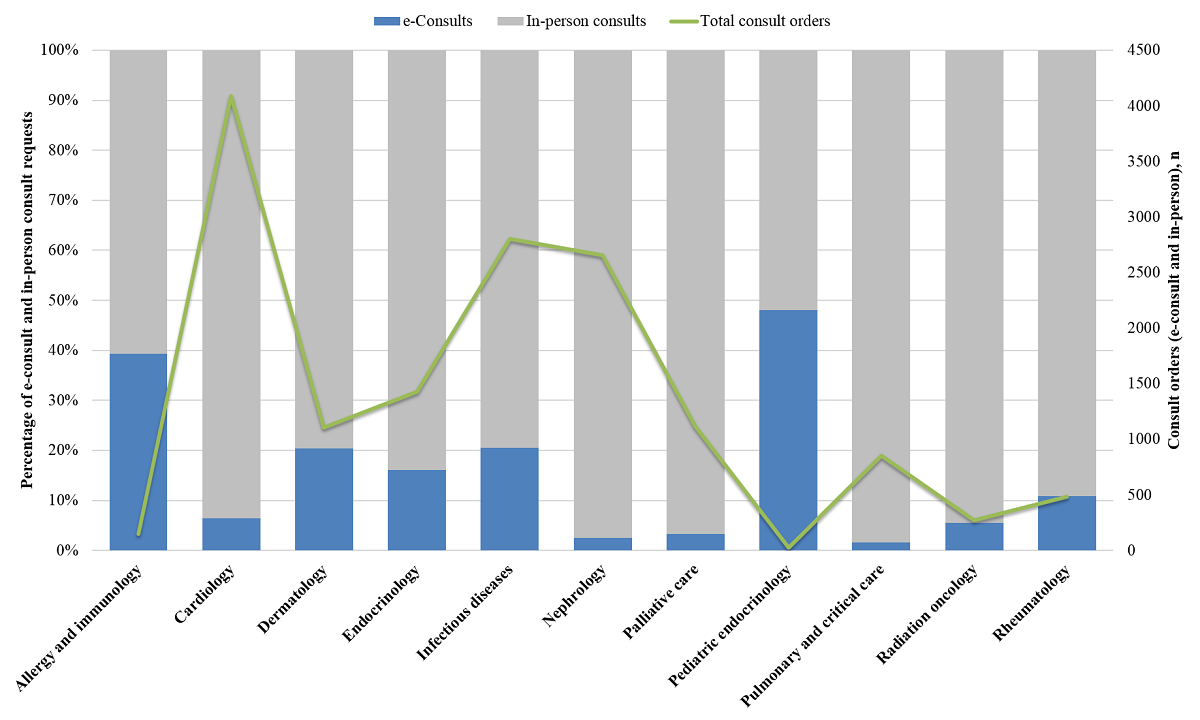
e-Consult and traditional in-person consult volume by specialty. Percentages of e-consults (blue) and traditional in-person consults (gray) requested by specialty are shown on the primary axis. Total consult orders (e-consult and traditional in-person consults) by specialty are shown on the secondary axis. e-consult: electronic consultation.

UCI’s e-consults service was launched in 11 total specialties (allergy and immunology, cardiology, dermatology, endocrinology, infectious diseases, nephrology, palliative care, pediatric endocrinology, pulmonary and critical care, radiation oncology, and rheumatology). Over a 2.5-year period, 1543 e-consults were requested out of 14,974 total consult orders (e-consult and traditional in-person consults) across the 11 participating specialties (Figure 2). Thus, the average proportion of consult orders requested as e-consults is 10.3%, although this proportion varied widely among participating specialties. The specialty with the lowest e-consult proportion was pulmonary and critical care, which had 1.5% (13/850) of total consult orders requested as e-consults, while the specialty with the highest e-consult proportion was pediatric endocrinology, which had 48% (12/25) of total consult orders requested as e-consults. However, with only 25 total consult orders, pediatric endocrinology had the fewest number of total consult orders of all participating specialties.

The most requested e-consult specialties were infectious diseases (which received 574/1543, 37.2% of the e-consult requests), cardiology (261/1543, 16.9% of the e-consult requests), endocrinology (229/1543, 14.8% of the e-consult requests), and dermatology (226/1543, 14.6% of the e-consult requests; Table 1). A total of 53.5% (826/1543) of e-consult requests across all participating specialties were addressed without the need for an in-person evaluation of the patient by the consulting team. The specialty with the fewest e-consult requests resolved electronically was pulmonary and critical care, which completed 0% (0/13) of requests electronically, while the specialty with the most e-consult requests resolved electronically was pediatric endocrinology, which completed 100% (12/12) of e-consult requests, without needing to physically examine the patient. However, both specialties had the smallest volumes of e-consult requests of all participating specialties.

**Figure 3:**
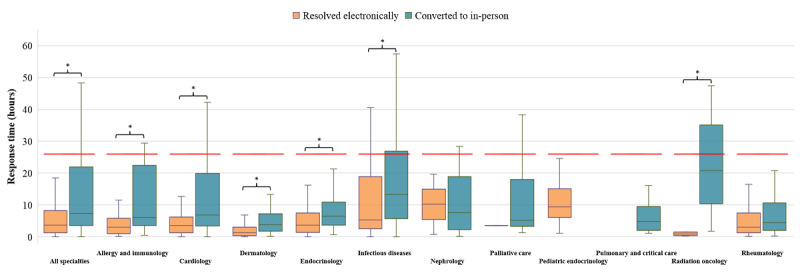
e-Consult response times by specialty. Boxplot of response times by specialty for e-consult requests resolved electronically (orange) and converted to in-person (teal). The box represents the IQR, with the top and bottom of box corresponding to the upper (third) and lower (first) quartiles, respectively. The horizontal line inside the box marks the median and the lines extending out from the box (whiskers) represent values within 1.5 times the IQR. For reference, the median response time for completion of a traditional in-person consult is marked by the dashed red line. *Indicates statistical significance.

We found that the overall median response time of e-consult requests resolved electronically was significantly lower than requests converted to an in-person consultation (Figure 3). The median time between ordering an e-consult and a specialist documenting recommendations in a consult note was 3.7 (IQR 1.3-8.2) hours across all specialties when resolved electronically, contrasted with 7.3 (IQR 3.6-22.0) hours when converted to an in-person consult (*P*<.001). Over half (6/11, 55%) of the participating specialties had significantly faster median e-consult response times for requests resolved electronically compared to requests converted to an in-person consultation. The specialties with the fastest e-consult response times were dermatology and radiation oncology, which had median response times of 1.3 (IQR 0.4-3.0) hours and 0.9 (IQR 0.3-1.5) hours when resolved electronically, respectively. However, radiation oncology had one of the smallest volumes of e-consult requests among participating specialties.

The overall response times of e-consult requests were much faster than the turnaround goal mandated by our institutional guidelines, which require a same-day response by 8 PM if the consult is ordered before noon or a response by the following morning if ordered after noon. For reference, the overall median response time for completion of a traditional in-person consult by the same 11 specialties during the same 2.5-year period is 25.8 (IQR 10.8-65.7) hours (Multimedia Appendix 1). Thus, regardless of whether an e-consult request was resolved electronically or converted to an in-person consult, e-consults significantly improved the turnaround times for inpatient consultations.

**Figure 4:**
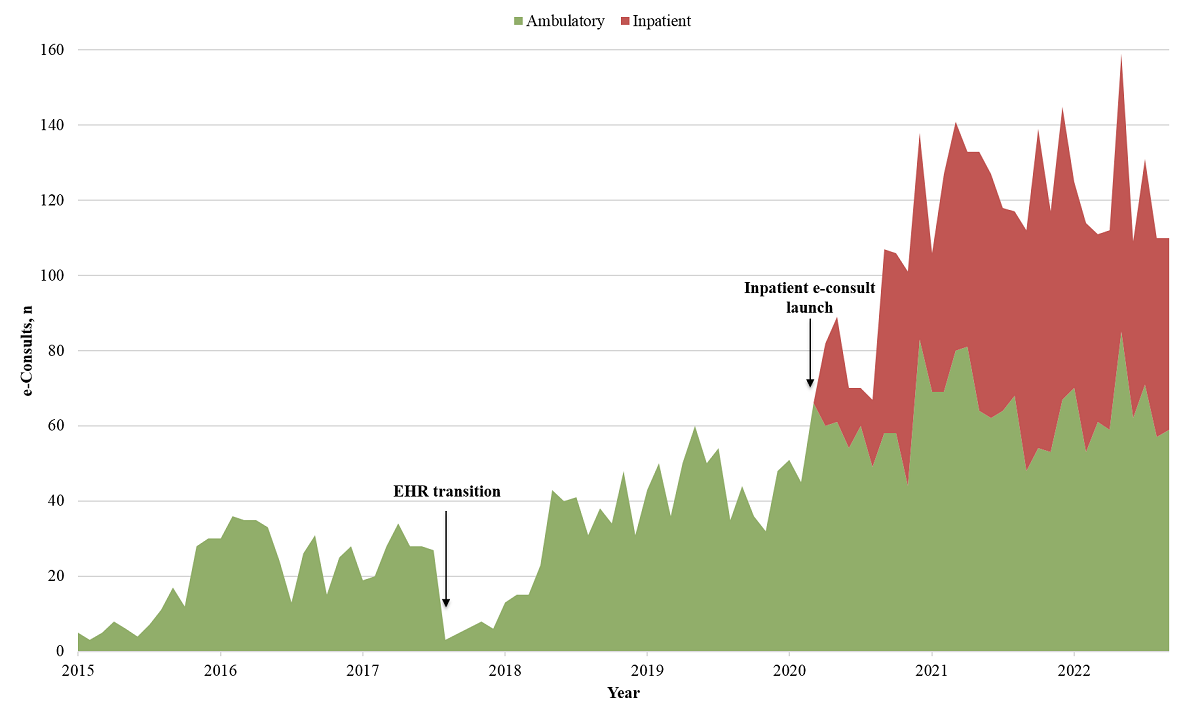
e-Consult volume over time. Monthly e-consult orders for ambulatory (green) and inpatient (red) settings at the University of California Irvine (UCI), 2015-2022. UCI transitioned from the Allscripts electronic health record platform to Epic in fall 2017. Launch dates for ambulatory and inpatient e-consults were April 2015 and July 2020, respectively. e-consult: electronic consultation; EHR: electronic health record.

The average volume of requests was 19 inpatient e-consults per month during the first 5 months that inpatient e-consults were live (Figure 4). Then, California experienced surges of COVID-19 cases throughout the pandemic and we saw corresponding increases in inpatient e-consults use. During the winter 2020 surge, the average volume of requests increased to 52 inpatient e-consults per month. Then, the Delta variant wave arrived in summer 2021, and the average volume of requests increased to 61 inpatient e-consults per month. When the Omicron variant wave emerged in winter 2021, the average volume of requests peaked at 75 inpatient e-consults per month. During a sustained wave in spring-summer 2022 driven by Omicron subvariants, the average volume of requests was 62 inpatient e-consults per month. After these surges subsided and COVID-19 cases declined, the use of inpatient e-consults remained at a high-level baseline with an average of 53 inpatient e-consults per month. Interestingly, similar patterns of increased e-consults use were observed in the ambulatory setting.

**Table 1:** Volume and outcomes of e-consult requests by specialty.

Specialty	e-Consult orders, N	Resolved electronically, n (%)	Converted to in-person, n (%)
All specialties	1543	826 (53.5)	717 (46.5)
Allergy and immunology	57	46 (80.7)	11 (19.3)
Cardiology	261	108 (41.4)	153 (58.6)
Dermatology	226	152 (67.3)	74 (32.7)
Endocrinology	229	140 (61.1)	89 (38.9)
Infectious diseases	574	335 (58.4)	239 (41.6)
Nephrology	68	2 (2.9)	66 (97.1)
Palliative care	36	1 (2.8)	35 (97.2)
Pediatric endocrinology	12	12 (100)	0 (0)
Pulmonary and critical care	13	0 (0)	13 (100)
Radiation oncology	15	4 (26.7)	11 (73.3)
Rheumatology	52	26 (50)	26 (50)

## Discussion

### Principal Findings

In response to the COVID-19 crisis, we successfully implemented an inpatient e-consult service that offered providers the option of requesting a subspecialty consultation for patients who are hospitalized and do not require an in-person evaluation by the specialist. Strong engagement by the clinical champions and technology partners in our steering committee, along with support from UCI’s leadership which provided us with dedicated IT, compliance, and billing teams, contributed to the successful design and implementation of our inpatient e-consults service. In addition, we were able to rapidly launch the service by leveraging our experiences with implementing e-consults in the ambulatory setting and capitalizing on existing infrastructure for inpatient consults. Instead of creating unique e-consult orders, configuring our existing inpatient consult order reduced the build components for our IT team, allowing us to quickly and effectively launch the inpatient e-consults service. Because we used workflows similar to those for traditional in-person consults, the processes for requesting and responding to inpatient e-consults were not new for providers. This strategy, along with provider familiarity with our well-established e-consults service in the ambulatory setting, likely helped to foster the adoption of inpatient e-consults. Although the COVID-19 crisis provided the key stimulus, these factors may have also contributed to the more rapid adoption of inpatient e-consults in comparison to the initial uptake of our ambulatory e-consults.

We found that the e-consult services helped to significantly reduce the response time for consults requested on patients who are hospitalized and do not require an in-person evaluation. In fact, the overall median response time of e-consult requests resolved electronically was approximately half of the response time for requests converted to an in-person consultation and nearly 7 times faster than the response time for traditional in-person consults. This time saving was critical during surges of COVID-19 cases when emergency departments and inpatient units were overwhelmed, leading to prolonged wait times for patients who were hospitalized to receive consultative care. e-Consults helped to streamline the inpatient consultation process and enabled the consulting team to promptly and efficiently provide recommendations on patients not needing a physical examination.

Although some diagnoses require in-person evaluation of the patient, lower complexity problems can be managed effectively using e-consults. Indeed, we found that over half of e-consult requests were addressed electronically without the need for an in-person evaluation of the patient by the consulting team. By reducing unnecessary in-person consultations, e-consults likely helped to limit the use of scarce PPE; minimize disease transmission; and free up specialists for other activities, such as examining patients with more complicated conditions and performing procedures. This improved resource use may also translate to potential cost savings associated with avoided in-person consultations and increased productivity. Future work should aim to analyze the cost-effectiveness of inpatient e-consults.

After the peaks of the COVID-19 crisis subsided in California, we discovered that provider use of inpatient e-consults persisted at a rate well above the precrisis levels. This sustained use implies positive provider experiences with the service and suggests a preference for e-consults when addressing lower complexity problems. Developing workflows for the inpatient e-consults service that were familiar to providers and significantly improving the turnaround times for inpatient consultations also likely helped to facilitate this sustainability. Thus, while case numbers and death rates associated with the COVID-19 pandemic have declined, e-consults continued to be an important part of our health care delivery.

Although relatively new, there have been a few reports of e-consults in the inpatient setting. The earliest examples involved the unexpected use of the ambulatory e-consult platform in the inpatient setting [13] and the design of an inpatient e-consult protocol that provided subspecialty consultations to inpatients at a remote hospital that lacked access to these clinical services [12]. Other reports described the feasibility and use of inpatient e-consults for only 1 specific specialty consulting service [10,11]. While 1 health system reported their implementation of an inpatient e-consult program in several specialties, provider adoption was temporary in response to the COVID-19 crisis [9]. Our experience with inpatient e-consults uniquely contrasts with these other health systems because we not only successfully implemented inpatient e-consults in multiple specialties but also demonstrated its sustained use beyond the pandemic.

### Limitations

Although anecdotal provider feedback has been positive, limitations to this study include the absence of a formal assessment of user experiences with the inpatient e-consults service. In addition, the volumes of e-consult requests and total consult orders were low for some specialties; thus, caution must be applied in the interpretation of results from these low-volume specialties. Nevertheless, we believe our unique development of inpatient e-consults is easily translatable to other institutions interested in implementing it and will lead to a positive user experience and greater use since we fit the e-consult process into already existing and common workflows of requesting a consultation. Additionally, although the implementation of our inpatient e-consults service was in a single academic health system, we successfully demonstrated that the use of e-consults in the inpatient setting is a promising approach to expediting patient care and reporting our experience in designing and implementing inpatient e-consults may provide guidance to other health systems considering similar telehealth models.

### Conclusions

Our implementation of e-consults in the inpatient setting highlighted an innovative use for e-consults in the era of COVID-19. It allowed for timely, efficient delivery of inpatient consultation services while reducing the unnecessary exposure of health care workers to potential infection. Consequently, inpatient e-consults likely helped to conserve precious PPE, minimize disease transmission, and enhance our ability to deal with surges in COVID-19 cases by expediting rapid assessment and management of lower complexity referrals. Although the COVID-19 emergency served as motivation to expand our ambulatory e-consults program to the inpatient setting, the service has become a vital component of our regular practices and will remain an essential part of our health care delivery, both in the ambulatory and inpatient settings, beyond the current pandemic, achieving sustainability and value.

## Appendix

Multimedia Appendix 1 Median (IQR) response times by specialty for traditional in-person consults, compared with median (IQR) response times for e-consults converted to in-person and e-consults resolved electronically.

## Abbreviations

e-consult: electronic consultation
EHR: electronic health record
PPE: personal protective equipment
UCI: University of California Irvine

## References

[1] Chen AH, Kushel MB, Grumbach K, Yee HF (2010). Practice profile. A safety-net system gains efficiencies through 'eReferrals' to specialists. Health Aff (Millwood).

[2] Keely E, Liddy C, Afkham A (2013). Utilization, benefits, and impact of an e-consultation service across diverse specialties and primary care providers. Telemed J E Health.

[3] Malagrino GD, Chaudhry R, Gardner M, Kahn M, Speer L, Spurrier BR, LaRusso NF (2012). A study of 6,000 electronic specialty consultations for person-centered care at The Mayo Clinic. Int J Pers Cent Med.

[4] Arora A, Fekieta R, Nouri Z, Carder D, Colgan MM, Fuhlbrigge A, Jackson SL, Collins S, Gleason N, Chen J (2022). Trends in utilization of electronic consultations associated with patient payer and language among US academic medical centers during the COVID-19 pandemic. JAMA Netw Open.

[5] Liddy C, Moroz I, Mihan A, Nawar N, Keely E (2019). A systematic review of asynchronous, provider-to-provider, electronic consultation services to improve access to specialty care available worldwide. Telemed J E Health.

[6] Vimalananda VG, Gupte G, Seraj SM, Orlander J, Berlowitz D, Fincke BG, Simon SR (2015). Electronic consultations (e-consults) to improve access to specialty care: a systematic review and narrative synthesis. J Telemed Telecare.

[7] Kuo S, Aledia A, O'Connell R, Rudkin S, Dangodara AA, Amin AN (2022). Implementation and impact on length of stay of a post-discharge remote patient monitoring program for acutely hospitalized COVID-19 pneumonia patients. JAMIA Open.

[8] Amin AN, Aledia A (2020). An eConsults program to improve patient access to specialty care in an academic health system. J Clin Outcomes Manag.

[9] Rikin S, Epstein EJ, Gendlina I (2021). Rapid implementation of inpatient eConsult programme addresses new challenges for patient care during COVID-19 pandemic. BMJ Innov.

[10] Mustafa SS, Staicu ML, Yang L, Baumeister T, Vadamalai K, Ramsey A (2020). Inpatient electronic consultations (E-consults) in allergy/immunology. J Allergy Clin Immunol Pract.

[11] Yagnik KJ, Saad HA, King HL, Bedimo RJ, Lehmann CU, Medford RJ (2021). Characteristics and outcomes of infectious diseases electronic COVID-19 consultations at a multisite academic health system. Cureus.

[12] Najafi N, Harrison JD, Duong J, Greenberg A, Cheng HQ (2017). It all just clicks: development of an inpatient e-consult program. J Hosp Med.

[13] Gupte G, Vimalananda V, Simon SR, DeVito K, Clark J, Orlander JD (2016). Disruptive innovation: implementation of electronic consultations in a veterans affairs health care system. JMIR Med Inform.

